# The Critical Gene Screening to Prevent Chromophobe Cell Renal Carcinoma Metastasis through TCGA and WGCNA

**DOI:** 10.1155/2022/2909095

**Published:** 2022-10-15

**Authors:** Cheng Yan, Yan Yang, Yunxiang Tang, Xiaojing Zheng, Bin Xu

**Affiliations:** ^1^Department of Oncology and Hematology, Chongqing Emergency Medical Center, Chongqing University Central Hospital, Chongqing 400014, China; ^2^Department of Nuclear Medicine, Chongqing Emergency Medical Center, Chongqing University Central Hospital, Chongqing 400014, China

## Abstract

Common chromophobe renal cell carcinoma (chRCC) has a good prognosis when cured by surgery. However, clinical practice shows that a small number of patients with chRCC will produce metastasis, and the prognosis after metastasis is poor. In this regard, we try to find potential biological targets to prevent CRCC metastasis. In this experiment, we analyzed the clinical traits and gene expression data of chRCC samples which were provided by the TCGA database by the WGCNA method. On this basis, we selected MEtan, a module with a significant positive correlation with the M phase of chRCC, for subsequent analysis. The MEtan module genes in the biological process of chRCC were mainly related to steroid metabolic process, cholesterol metabolic process and STEM cell differentiation. KEGG analysis showed that these genes were mainly enriched in cancer-related signaling pathways, such as Neuroactive Ligand−receptor interaction, cAMP signaling pathway, and Wnt signaling pathway. Subsequently, we mapped the PPI interaction network and screened the key gene beta-arrestin 2 (ARRB2). Expression analysis showed that there was a significantly increased expression of ARRB2 in chRCC patients in comparison to the normal group. Expression survival analysis indicated that ARRB2 was inversely associated with overall survival. We firmly believe that the key genes identified in this study would be able to provide new clues and research basis for the treatment of chRCC.

## 1. Introduction

Over 400,000 cases of renal cell carcinoma (RCC) are diagnosed each year in the world, making it one of the most common renal malignancies [1]. Pathologically, RCC is divided into three types: clear cell renal cell carcinoma (ccRCC), papillary carcinoma (pRCC), and chromophobe carcinoma (chRCC). ChRCC is the third subtype of RCC recognized by the World Health Organization (WHO) in 2016 [2]. An estimated 5-10% of all kidney cancers are chRCC, which are equally common in men and women, with a higher incidence in those aged 50-60 [3–5]. ChRCC behaves differently than other types of renal cell carcinomas. Recent statistics indicate an increase in chRCC incidence [6, 7]. Patients with chRCC may present with hematuria or tumor compression symptoms, and a few show diffuse growth and invasion of the perirenal region [8, 9].

A large number of clinical practices have shown that chRCC is usually cured by surgery, and the prognosis of patients is good, with 5-year survival rates of 78-100% and 10-year survival rates of 80-90%; however, there are still 5-10% of patients with chRCC who will develop metastases [10, 11]. Approximately 14 percent of patients with metastatic RCC will survive five years, similar to those with definite metastatic chRCC [12]. Therefore, an in-depth study of the genes related to the pathogenesis of chRCC will comprehensively explain the pathogenesis and disease progression of chRCC, which is of great significance for its treatment and prevention.

There is currently no more comprehensive tumor gene expression profile database than the Cancer Genome Atlas (TCGA), which is distinguished by its large sample size and rich clinical information [13]. An analysis of gene-phenotype relationships called Weighted Gene Coexpression Network Analysis (WGCNA) has gained popularity for its ability to investigate complex relationships between genes and phenotypes. With the WGCNA method, researchers are able to transform gene expression data into coexpression modules and provide insights into signaling networks that may be responsible for the phenotypic characteristics of the object of interest [14].

Data from gene chips related to chRCC disease were integrated and analyzed using bioinformatics technology: GO and KEGG pathway enrichment analyses were performed first to filter out the differential genes; then, we commenced WGCNA to analyze the clinical characteristics and gene expression data of chRCC samples provided by TCGA database, and made PPI interaction network to find the key genes in the pathogenesis and development of renal chromophobe cell carcinoma; the final step of our study was to investigate the survival of the genes mined to reconstruct the mechanism of renal chromophobe cell carcinoma.

## 2. Materials and Methods

### 2.1. Data Capturing

The TCGA Datasets (https://www.cancer.gov/about-nci/organization/ccg/research/structural-genomics/tcga) in the database were accessed with the keyword of chromophobe takes cell carcinoma to search, and the genome data of renal color cell cancer was downloaded. The data included 65 chromophobe cell carcinoma tumor samples and 25 normal tissue samples.

### 2.2. DEG Capturing

Standardizing and analyzing renal chromophobe cell carcinoma datasets were done by using the DESeq2 algorithm in R software. A difference factor (log2) absolute value higher than 1 was used to screen upregulated genes. Ggplot2 software package was used for data visualization.

### 2.3. WGCNA Analysis

WGCNA provides R functions that help analyze gene expression data using weighted correlation networks.

The source code and other materials for this R package are available for free at http://genetics.ucla.edu/labs/horvath/CoexpressionNetwork/Rpackages/WGCNA. Our coexpression network was built using the WCCNA R package. In the first step, clustering the samples was performed to identify any outliers. Next, the coexpression network was constructed using the automatic network construction function. Coexpression similarity is proposed to reckon the adjacency with the R function pickSoftThreshold.

### 2.4. Module-Trait Relationship Analysis

The corresponding gene modules were sorted according to the WGCNA modules; then, the ME for each module was calculated and correlated with clinical parameters, with statistical significance defined as *P* < 0.05.

### 2.5. Differentially Expressed Genes Enriched in GO and KEGG

DAVID database (DAVID; https://david.ncifcrf.gov) was used to analyze GO enrichment and KEGG pathway enrichment of significant different genes screened. The R software and clusterProfiler package were used for annotation and visualization, and a *P* value less than 0.05 was considered statistically significant.

### 2.6. Screening for Hub Genes in the PPI Network and Construction of a Protein-Protein Interaction Network

Interactions between proteins were identified and predicted using the STRING database (https://string-db.org/). Protein-protein interaction (PPI) networks were constructed using STRING for analysis of differentially expressed genes, and screening for hub genes in the STRING PPI network was performed using the Cytohubba plug-in in Cytoscape software.

### 2.7. Key Gene Survival Analysis

R software was used to analyze the survival of the selected key genes, and an analysis to Kaplan-Meier survival curves was carried out to determine the relationship between the key genes and renal chromophobe cell carcinoma recurrence. An evaluation of the survival difference between key genes was conducted via a log-rank test and the overall survival rate for renal chromophobe cell carcinoma patients was *P* < 0.05, deemed significant.

## 3. Results

### 3.1. Differentially Expressed Genes Analysis

**Figure 1:**
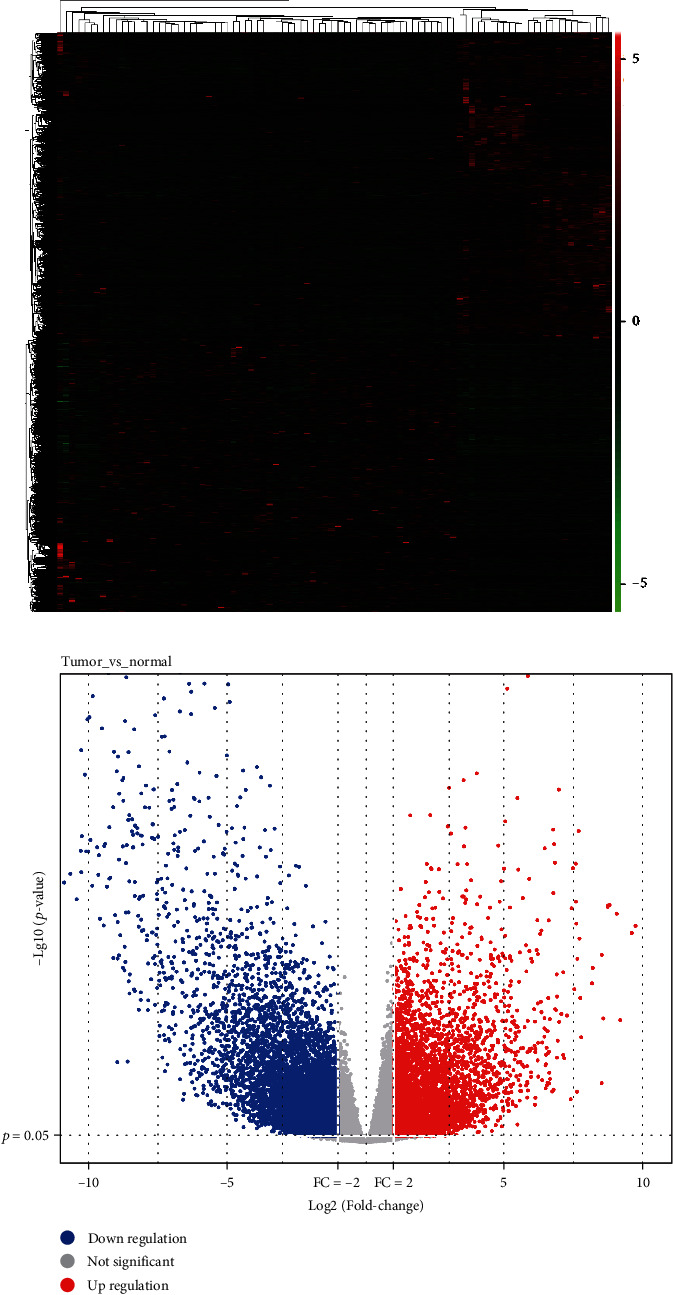
DEG analysis. (a) heat maps; (b) volcanic map.

**Figure 2:**
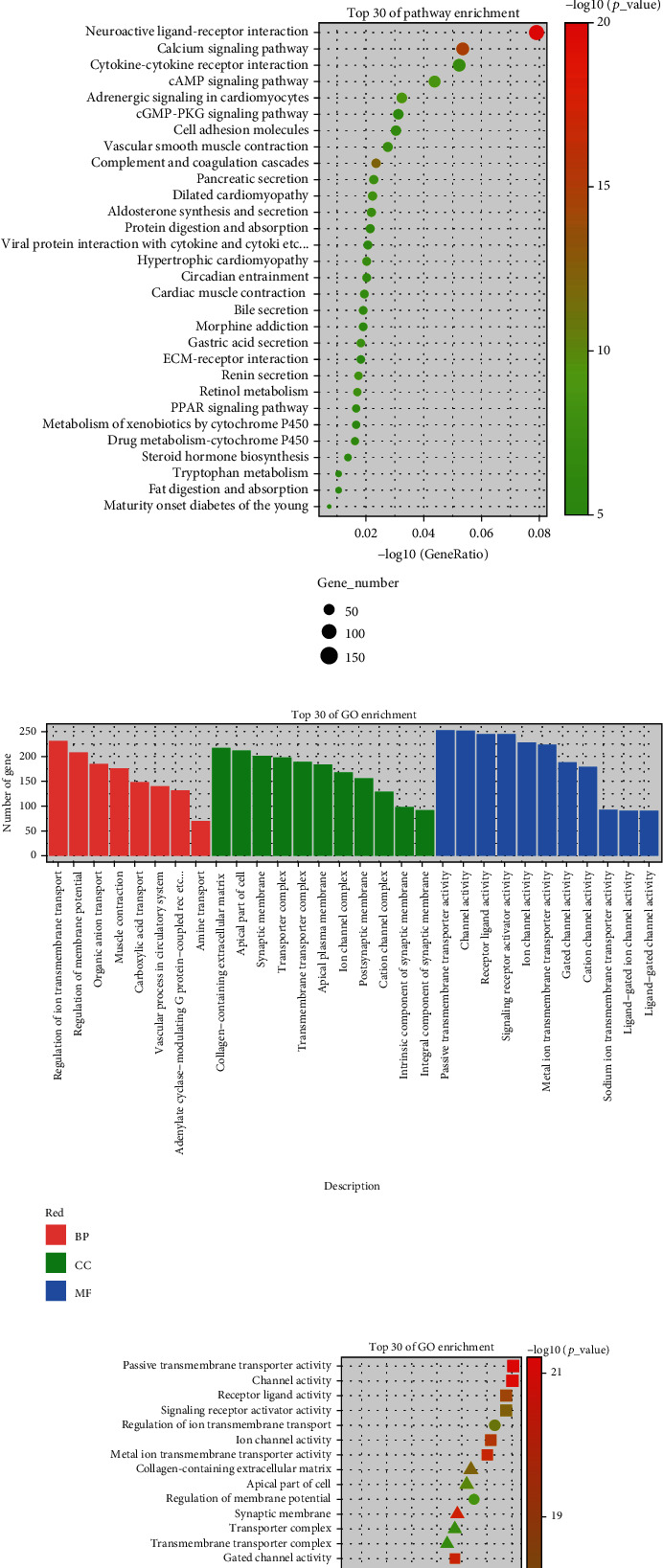
DEG pathway enrichment analysis. (a and b) KEGG enrichment analysis; (c and d) GO enrichment analysis.

An analysis of the transcriptome data from TCGA database was conducted on 65 chromophobe cell carcinoma tumor samples and 25 normal tissue samples. The DESeq2 tool identified 13472 DEGs, of which 6066 were upregulated and 7406 were downregulated (Figures 1(a) and 1(b)). We ran KEGG enrichment analyses on the top 30 DEGs with a *P* < 0.05 standard, and results showed that they mainly concentrated on pathways of cAMP, Cytokine−cytokine receptor interaction, Calcium, and Neuroactive Ligand−receptor interaction, etc. (Figures 2(a) and 2(b)). A GO enrichment analysis identified three biological processes associated with DEGs: ion transmembrane transport, membrane potential regulation, and organic anion transport; cell composition included an apical area, extracellular matrix containing collagen, and synaptic membrane; there were several molecular functions that were examined, such as passive transmembrane transporter activity, channel activity, receptor ligand activity, and signaling receptor activator activity (Figures 2(c) and 2(d)).

### 3.2. A Weighted Coexpression Network Analysis

**Figure 3:**
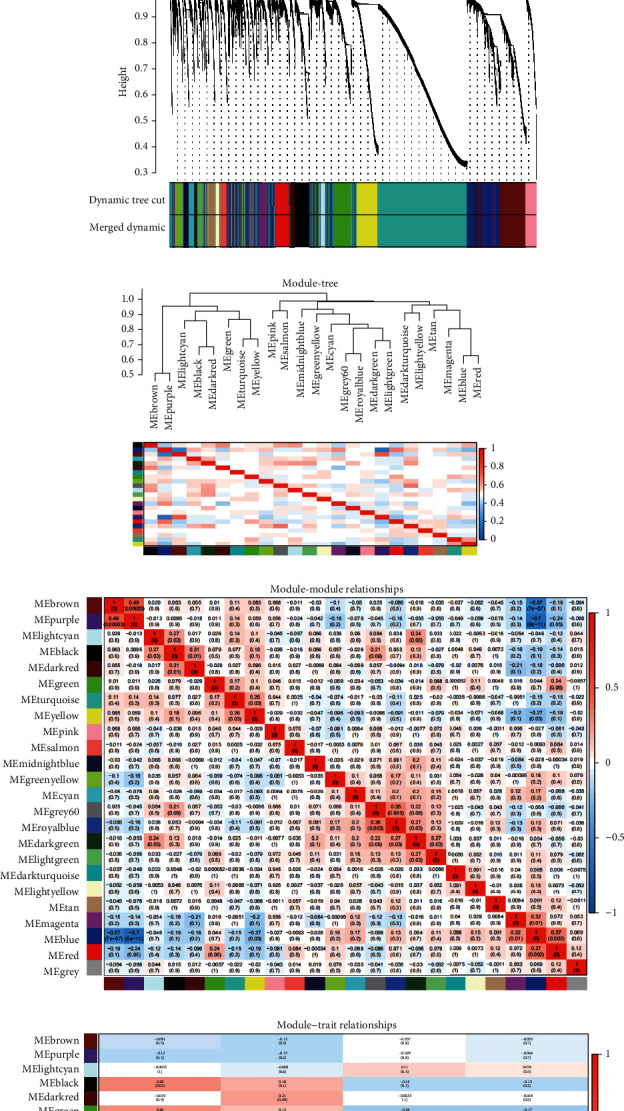
DEG analysis was carried out by weighted gene coexpression network analysis (WGCNA) method and gene cluster tree analysis of modular feature genes. (a) Scale-free exponential analysis to various soft threshold powers (*β*). (b) The color of the module represented by each dendrogram of the cluster module of the DEG (top) and the color band (bottom). (c) Clustering dendrograms of different genes based on topological overlap, and the colors assigned to the corresponding modules. (d) Correlation analysis onto different modules. (e) Correlation analysis onto modules and traits. ME: module characteristic gene.

Our first step in constructing the WGCNA network was to calculate the soft threshold power *β*. It was determined that the soft threshold power was 3-; the scale independence was 0.9, and the average connectivity was relatively high (Figure 3(a)). Our gene network construction and module identification was done via the WGCNA R package's one-step network construction function.  Figure 3(b) displayed the color-coded coexpressed gene modules identified via WGCNA method, where the grey by default was those genes that could not be classified into any module. It was found that these modules could be classified into two categories and 23 subclasses, and that there was correlation amid these modules (Figures 3(c) and 3(d)). A second purpose of WGCNA is to analyze the correlation between modules and clinical parameters (*R* value). Analysis to the correlation amid the module genes and chRCC showed that the modules MEblack, MEgreen, and MEtan were significantly positively correlated with the M phase of chRCC, and the correlation coefficients *r* were 0.28, 0.26, and 0.32, respectively (*P* < 0.05, Figure 3(e)). According to Figure 3(e), MEdarkred was positively correlated with T phase, and the correlation coefficient *r* was 0.21 (*P* < 0.05). Figure 3(e) illustrated a positive correlation between MEdarkred and T phase (*r* = 0.21; *P* < 0.05).

### 3.3. Module MEtan Gene Functional Enrichment Analysis

**Figure 4:**
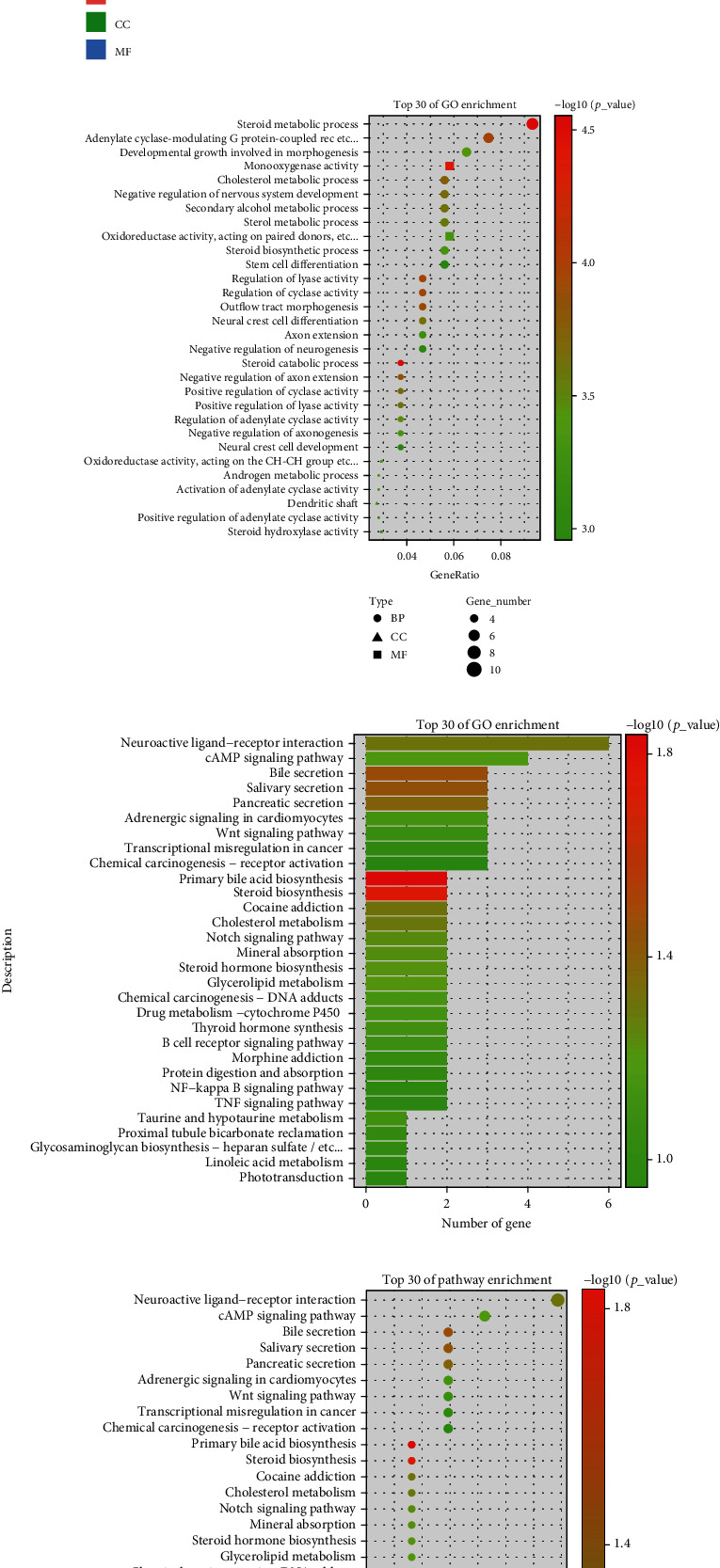
GO and KEGG analysis for module genes. (a and b) GO-term analysis and (c and d) KEGG enrichment analysis of MEtan module.

The above analysis led us to select MEtan for further analysis, because it has a significant positive correlation with the M phase of chRCC. GO analysis revealed that steroid metabolism, cholesterol metabolism, and stem cell differentiation were the top chRCC biological processes of MEtan module genes (Figures 4(a) and 4(b)). Genes enriched in cancer-related pathways, such as Neuroactive–Ligand receptor interaction, cAMP signaling pathway, and Wnt signaling pathway, were identified in KEGG analysis. (Figures 4(c) and 4(d)).

### 3.4. Screening to Hub Genes

**Figure 5:**
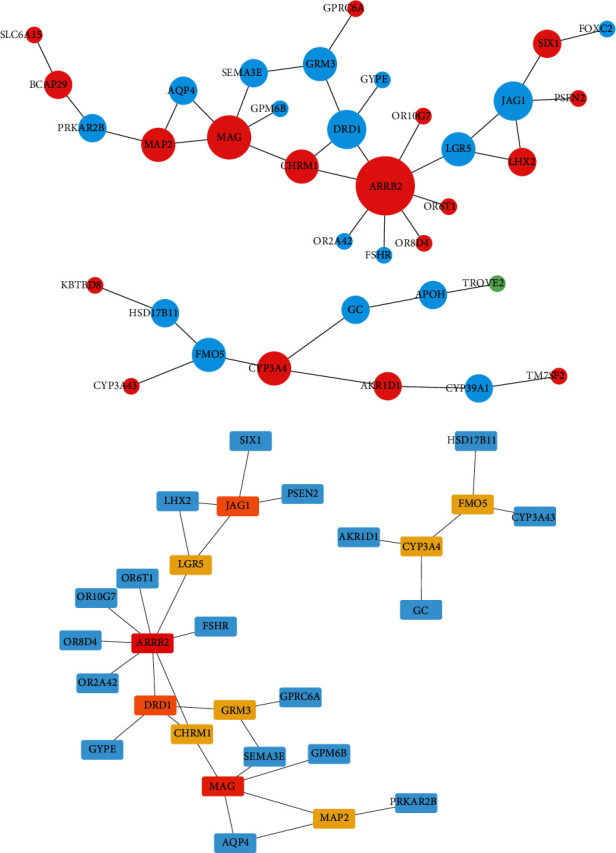
Construction of PPI network and screening to the key genes. (a) Protein-protein interaction network construction of differentially expressed genes in the MEtan module and (b) Cytoscape analysis. Note: The darker the color, the stronger the correlation.

With the help of the STRING online database and Cytoscape software, DEGs from MEtan modules were analyzed, and PPI networks were constructed in order to identify key genes. Cytoscape's CytoHubba plugin was used to screen the PPI network for key genes. MAG, CHRM1, and ARRB2 were in the center of the 36 nodes in the PPI network for module MEtan (Figure 5(a)). Finally, ARRB2 and MAG were the main genes we screened out (Figure 5(b)).

### 3.5. Survival Analysis

**Figure 6:**
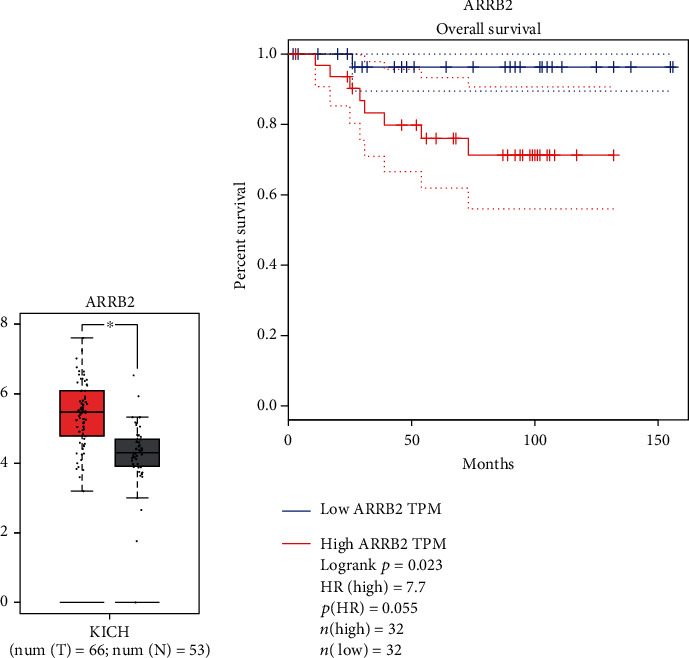
Overall survival analysis. (a) expression analysis to ARRB2 the key gene in MEtan module; (b) correlation analysis to ARRB2 expression and chRCC patients' overall survival; KICH: kidney chromophobe.

In contrast to the normal group, chRCC patients expressed significantly more ARRB2 than do normal individuals (Figure 6(a), *P* < 0.05). Kaplan-Meier survival curves were constructed to analyze chRCC ‘s overall survival rate. All chRCC samples were divided into high expression group and low expression group of key genes, and compared with the median value of key genes; according to expression survival analysis, ARRB2 was negatively correlated with overall survival (Figure 6(b), *P* > 0.05).

## 4. Discussion

ChRCC develops from dark cells in the collecting duct epithelium of the kidney [15]. There was 89.3% recurrence-free survival (RFS) and 93% cancer-specific survival (CSS) rates for chRCC after 5 years [16]. Metastatic disease accounts for only 6% of chRCC patients [17]. However, patients with metastatic chRCC illness have a poor prognosis, who more frequently show nodular characteristics and have a low incidence of treatment response [18, 19]. On postoperative follow up, Geramizadeh et al. found that only 20 (16%) of 123 CRCC patients progressed (local recurrence, metastasis, or death) [20]. Therefore, an in-depth study to related genes coexpressed in various stages and links of chRCC and discovery of genes that play a crucial regulatory role in its occurrence; furthermore, the development of the disease is indispensable for understanding its mechanism and improving treatment measures.

An in-depth analysis of the key genes involved in renal chromophobe cell carcinoma development and progression was undertaken in this study. A total of 13466 differentially expressed genes of renal chromophobe cell carcinoma were screened and mined by searching TCGA database, among which 6066 genes were upregulated and 7406 genes were downregulated. Several of these DEGs converged on the signaling pathways involving cAMP, cytokine-cytokine receptor interaction, calcium signaling pathway, and Neuroactive Ligand–receptor interaction.

An advantage of the WGCNA method is that it explores the association between clinical traits and coexpression modules, with higher reliability and biological significance [21]. TCGA database samples were analyzed through the WGCNA method to analyze clinical traits and gene expression data. According to the results, the modules MEblack, MEgreen, and MEtan were positively correlated with the M phase of chRCC; the module MEdarkred was positively correlated with the T phase of chRCC; moreover, MEsalmon is also positively correlated with the stage of chRCC. MEtan, which has a significant positive correlation with the M phase of chRCC, was selected for further analysis. Main chRCC biological processes of MEtan module genes include steroid metabolic process, cholesterol metabolic process, and STEM cell differentiation; besides, KEGG analysis revealed that these genes were primarily enriched in cancer-related signaling pathways such as Neuroactive Ligand−receptor interaction, cAMP signaling pathway, and Wnt signaling pathway.

Studies have shown that cyclic adenosine monophosphate (cAMP) plays an important role in controlling cell proliferation [22]. A total of 19 secreted glycoproteins make up the Wnt family, which regulates cell proliferation, differentiation, survival, migration, and stem cell self-renewal [23, 24]. There is an association between high Wnt1 expression in ccRCCs, increased tumor diameter, and more advanced stages [25]. A significant increase in WNT10A expression was also observed in RCC cells and tissues, and it plays an oncogenic role [26].

With the help of the STRING online database and Cytoscape software, DEGs from MEtan modules were analyzed, and PPI networks were constructed in order to identify key genes, and the key gene was ARRB2. In comparison with the normal group, ARRB2 expression was significantly higher in chRCC patients. ARRB2 expression was negatively correlated with overall survival, according to an expression survival analysis. There is a widespread expression of Arrb2, a multifunctional protein that regulates the desensitization and intracellular transport of G protein-coupled receptors (GPCRs) [27, 28]. Furthermore, Arrb2 is involved in a variety of signaling pathways, including those that involve extracellular signal-regulated kinases (ERK) and protein kinase B (Akt) [29, 30].ARRB2 has been shown to be involved in the metastasis of a variety of cancer cells. Defective SUMOylation of ARRB2 inhibits the migration of breast cancer cells and has been shown to be involved in ARRB2-dependent metabolic regulation of breast cancer cells [31]. ARRB2 plays a negative regulatory role in glioma growth, invasion, and metastasis by reducing HIF-1*α* expression and inhibiting angiogenesis [32]. It was found that inhibition of ARRB2 expression reduced local and metastatic RCC tumor growth [33]. In summary, ARRB2 may consider as a target for therapeutic intervention against tumour development and metastasis in the studies of future. This study provides a reference for the clinical application of ARRB2 as a prognostic biomarker and potential therapeutic target, and we will enrich its mechanism of action in chRCC through more experiments in the future.

## 5. Conclusion

This study screened TCGA databases for genes associated with chRCC occurrence and development and discussed key genes related to chRCC. A possible therapeutic target and prognostic marker for renal chromophobe cell carcinoma may be ARRB2. However, since there have been no studies on the gene level related to chRCC, there is an urgent need for more research into the biological role of chRCC in renal chromophobe cell carcinoma pathogenesis, so that new clues and directions will be offered for the treatment of renal chromophobe cell carcinoma.

## Data Availability

The data that support this study are in the article.
